# Determining efficacy of monitoring devices on ceramic bond to resin composite

**DOI:** 10.4317/medoral.18200

**Published:** 2012-05-01

**Authors:** Estrella Osorio, Fátima S. Aguilera, Raquel Osorio, Franklin García-Godoy, Miguel A. Cabrerizo-Vilchez, Manuel Toledano

**Affiliations:** 1 Professor, Department of Dental Materials, School of Dentistry, University of Granada, 18071 Granada, Spain; 2 Professor, Bioscience Research Center, College of Dentistry, University of Tennessee, Memphis, 38163 Tennessee, USA; 3 Professor, Applied Physics Department, University of Granada, 18071 Granada, Spain

## Abstract

Objectives: This paper aims to assess the effectiveness of 3D nanoroughness and 2D microroughness evaluations, by their correlation with contact angle measurements and shear bond strength test, in order to evaluate the effect of two different acids conditioning on the bonding efficacy of a leucite-based glass-ceramic to a composite resin. Study Design: Ceramic (IPS Empress) blocks were treated as follows: 1) no treatment, 2) 37% phosphoric acid (H3PO4), 15 s, 3) 9% hydrofluoric acid (HF), 5 min. Micro- and nano-roughness were assessed with a profilometer and by means of an atomic force microscopy (AFM). Water contact angle (CA) measurements were determined to assess wettability of the ceramic surfaces with the asixymetric drop shape analysis contact diameter technique. Shear bond strength (SBS) was tested to a resin composite (Z100) with three different adhesive systems (Scotchbond Multipurpose Plus, Clearfil New Bond, ProBOND). Scanning electron microscopy (SEM) images were performed. Results: Nanoroughness values assessed in 50x50 μm areas were higher for the HF group, these differences were not detected by profilometric analysis. HF treatment created the nano- roughest surfaces and the smallest CA (p<0.05), producing the highest SBS to the composite resin with all tested adhesive systems (p<0.05). No differences existed between the SBS produced by the adhesive systems evaluated with any of the surface treatments tested. Conclusions: Nano-roughness obtained in a 50x50 µm scan size areas was the most reliable data to evaluate the topographical changes produced by the different acid treatments on ceramic surfaces.

** Key words:**Dental ceramic, acid etching, bonding efficacy, resin composite, adhesive systems, contact angle, roughness.

## Introduction

Ceramics have been traditionally preferred as a dental restorative material because of their aesthetic quality and excellent biocompatibility. Self adhesive resin cements that rely on a single step application have been proposed for luting ceramic restorations to the tooth substrate (1). Long-term retention of the obturation depends primarily on the strength and durability of the bond between the luting composite resin, the tooth and the porcelain substrates to prevent fracture, marginal discoloration, and secondary caries (2).

To achieve the bond between ceramic and composite resin, the porcelain surface may be modified chemically or mechanically to promote surface roughness and/or reactivity of the porcelain to the luting agent (3). Acid etching is commonly the method aimed to achieve an irregular surface area for bonding (4). The glassy matrix is selectively removed and crystalline structures are exposed (5), increasing the surface energy and the wettability of the ceramic substrate (6). Hydrofluoric acid is commonly used to etch the bonding surface of porcelain restorations as the most effective etch on ceramic surfaces (3). Phosphoric acid was also investigated. However, its validity in achieving adequate bonding is still controversial, because it exhibited a slight etching effect (2,3).

In general, adhesive bonding is dependent on the surface energy and wettability of the adherent by the adhesive (4). This is one of the most important issues in adhesion, dealing with surface-free energy of ceramic and depends on surface roughness, waviness and ceramic chemical composition (7).

Surface roughness plays an important role in the ceramic adhesion but the evaluation is complex because many factors affect the roughness values. Most of the microscopic details can be lost, just because the experimental technique does not allow analysing them or they are overshadowed by their mathematical expressions (8). Conventional surfaces profilers used for roughness measurements can lead to incorrect measurements (9). Atomic force microscopy (AFM) has become an increasingly important tool for the measurements of surface roughness. Topography can be quantified at extremely high lateral and vertical resolutions. When the adhesion process involves the interaction of micron and sub-micron features, the knowledge of that low-scale roughness is fundamental, avoiding the use of more macroscopic roughness estimations, which could be heavily influenced by higher-scales roughness components (e.g. waviness), which are less relevant for process involving microscopic features (8).

The introduction in 1994 of the micro tensile bond strength test (10) allowing measurements of the tensile bond strength on very small surfaces opened the research to regional differences within dentin, and producing many specimens from the same tooth, it was thought that the conventional shear bond strength test would diminish (11). In spite of the increased popularity of the “micro” bond strength tests and the criticism endured by the conventional tensile and shear methods, the number of articles using “macro” tests published in recent years remains high, meaning that a lot of the available data on dental adhesion still comes from mechanical tests performed in specimens with large bonded areas. This is justified because they are easy to perform, requiring minimal equipment and specimen preparation (12).

No previous studies were found by using 3D nanorougness evaluation, 2D micro-roughness examination, Contact angle (CA) measurements and shear bond strength (SBS) test (macro-shear bond strength) to evaluate the effect of surface treatments on the bonding efficacy of ceramic to resin. The aim of this study was to evaluate the effectiveness of 3D nanoroughness and 2D micro roughness evaluations, through their correlation with contact angle measurements and shear bond strength test, in order to evaluate the effect of two different acids conditioning on the bonding efficacy of a leucite-based glass-ceramic to a composite resin.

The null hypothesis of the present study is that the 3D nano-roughness assessments do not differ to the 2D micro-roughness results from the two different acid-treatments applied on the ceramic surface. Contact angle and shear bond strength results are in accord with both roughness evaluation methods.

## Material and Methods

2.1. Specimen preparation

Seventy six ceramic cylinders of a hot-pressed leucite-based glass-ceramic, IPS Empress (Ivoclar-Vivadent, Schaan, Liechtenstein), were embedded in auto-curing acrylic resin (Special Try, Dentsply-DeTrey, Milford, USA). The exposed porcelain surfaces were mounted on a circular grinder (EXAKT-Apparatebau, Otto Herrman, Nortedst, Germany) and polished with sand SiC paper of 500, 800, 1000 and 1200 grit with water-proof to provide uniform flat polished ceramic surfaces.

Specimens were randomized into three groups: 1) no treatment, 2) treated with phosphoric acid gel (Total Etch, Ivoclar Vivadent, Schaan, Liechtenstein) (37% H3PO4) for 15 s, and 3) conditioned with hydrofluoric acid (Panreac Química S.A., Barcelona, Spain) (9% HF) for 5 min with constant agitation. After each etching process, the surfaces were rinsed with distilled water for 15 s. Acid pH values were measured with a pH meter (Micro pH 2000, Crison Instrument S.A., Alella, Barcelona, Spain).

2.2. Contact angle measurements

The axisymmetric drop shape analysis contact diameter (ADSA-CD) technique was used for contact angle measurements. Twelve consecutive 0.3 μl drops of deionized water were placed on the ground specimens, phosphoric conditioned samples and hydrofluoric acids etched ceramic surfaces with an Eppendorf micropipette (Eppendorf Scientific Inc., Westbury, NY, USA), and the observed contact angles were measured.

2.3. 2D Micro-roughness determinations (Ra).

Surface micro-roughness of each sample was measured with a profilometer (Mitutoyo Surftest 201, Tokyo, Japan) after performing the contact angle measurements in all groups. For each specimen, twelve diametric measurements (four scores for three different directions) were made and the average values (Ra) were calculated in microns with a cutoff value of 0.8.

2.4. 3D Nano-roughness measurements (sRa), Atomic Force Microscopy (AFM) images and graphs of profiles.

Six additional ceramic disks (1 mm thick) were obtained using a slow-speed water-cooled diamond saw (Buehler Instruments, Lake Bluff, IL, USA). All samples were metallographically polished to 1/4 µm diamond paste and were randomized into three groups as previously described. Each treatment group was evaluated under an Atomic Force Microscope (Multimode Nanoscope IIIa, Digital Instruments, Veeco Metrology Group, Santa Barbara, CA, USA). Digital images were taken in air. The tapping mode was performed using a 1-10 Ohm-Cm phosphorus (n) dopes Si tip (at 50 µm). Changes in vertical position provided the height of the images, registered as bright and dark regions. Fields of view at 5 x 5 µm and 50 x 50 µm scan sizes were considered for each disc at a data scale of 1504 µm and recorded with a slow scan rate (0.1 Hz). Five measurements were performed for each surface using a standardized square spot (2 x 2 µm and 10 x 10 µm respectively). Roughness was expressed by a numeric value (sRa) (nm) with specific software (Nanoscope V530R35R, Digital Instruments, Veeco Metrology Group, Santa Barbara, CA, USA). Graphs of roughness profiles (4.5 section) were acquired for treated and untreated ceramic surfaces.

2.5. Shear Bond strength (SBS) test 

Shear bond strength was determined according to ISO/TS 11405:2003[14] ISO/TS 11405:2003 (E): Dental materials—testing of adhesion to tooth structure. 2nd ed.; 2003.. Sixty porcelain specimens were randomly divided in two groups, followed each groups was separated into three subgroups. One system adhesive was tested in each subgroup after each acid treatment. Tested adhesive systems were: ScotchbondTM Multipurpose Plus (SBMP) (3M ESPE, St. Paul, USA), Clearfil New Bond (Clearfil) (Kuraray Europe GmbH, Germany) and ProBONDTM (Probond) (Dentsply/DeTrey, Milford, USA) ( Table 1). The specimens were embedded in die stone in one half of a Watanabe jig (13). The bonding agents were applied according to the manufacturers’ directions.

**Table 1:**
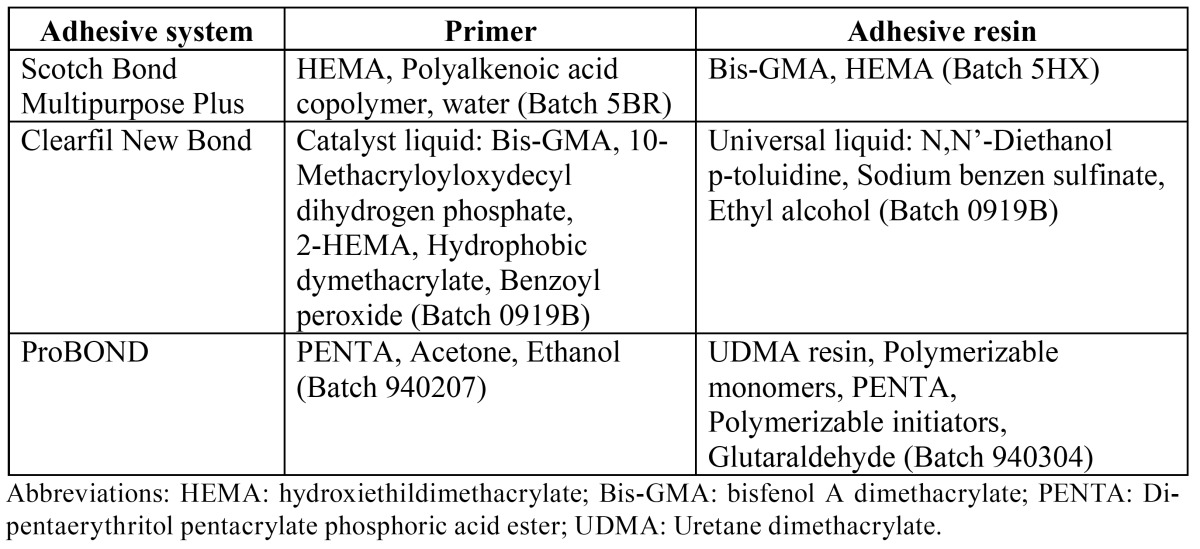
Adhesive systems evaluated.

The remaining half of the Watanabe jig (large hole plate) was then secured with a mylar mask with a 4 mm diameter contact hole containing the bonding agent. The exposed ceramic surface was aligned to the other shear plate. Z-100 resin composite (3M ESPE, St. Paul, USA) was inserted 1-1.5 mm increments and cured for 40 s with a Spectrum 800 curing unitn. Curing light output was monitored with a Demetron Curing Radiometer-Model 100 (Demetron Research Corporation, Danbury, CT, USA) to insure a constant value of at least 500 mW/cm2. The total composite resin bulk thickness was approximately 3 mm.

All specimens were stored in distilled water at 37oC for 24 h. Shear bond strengths (MPa) were obtained using an Instron testing machine (Instron Inc., Canton, MA, USA) at a crosshead speed of 0.75 mm/min. Failure modes were evaluated by a single operator under a stereo microscope (Olympus SZ-CTV, Olympus Co., Tokyo, Japan) at 40x magnification and classified as cohesive, adhesive or mixed failures.

2.6. SEM analysis

Representative specimens from each groups (polished, phosphoric treated and HF conditioned) were desiccated, gold-coated, and observed with a scanning electron microscope (SEM) (Zeiss DSM-950, Karl-Zeiss, Germany) at an accelerating voltage of 20 kV to examine the morphology of the studied surfaces.

2.7. Statistical analysis

Means of roughness and contact angle were compared using the Student’s t-test. Statistical significance was considered at a confidence level of 95%. Data of shear bond strength were analyzed with a two-way ANOVA (etching treatment and bonding system as independent variables) and Student-Newman-Keul’s multiple range tests (p<0.05).

## Results

**Figure 1:**
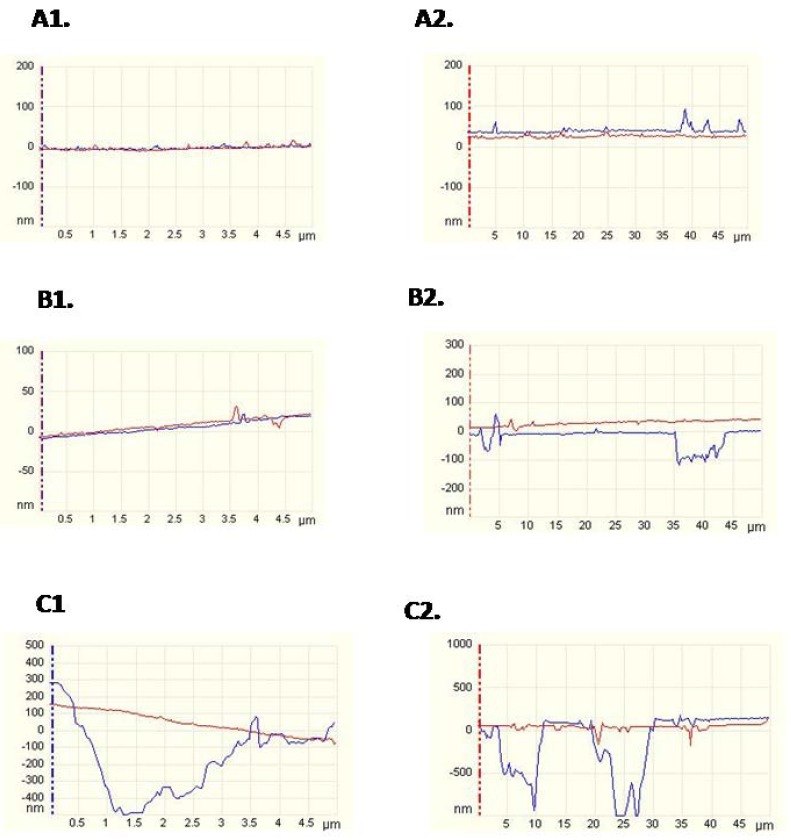
Roughness profile sections of no treated porcelain surfaces (flat line) and treated ceramic surfaces (waving line) at the different ceramic surfaces: A) no treatment, B) 37% phosphoric acid for 15 s., C) 9% HF for 5 min. and different scan sizes: 1) 5x5 µm and 2) 50x50 µm.

( Table 2) showed means and standard deviations of 2D micro-roughness (Ra) and 3D nano-roughness (sRa). (Fig. 1) exhibited the roughness profiles obtained along 5 µm and 50 µm by AFM. Roughness of ceramic surfaces were affected by the etching treatments (p<0.001). HF conditioned group exhibited the highest roughness means. 2D micro and 3D nano-roughness with a 5x5 µm scan size of the phosphoric acid group was significantly similar to polished ceramic (Fig. 1: A1 and B1). In 50 x 50 microns 3D nanoroughness measurements, phosphoric acid group exhibited a rougher surface than the polished ones (Fig.1: A2 and B2).

**Table 2:**
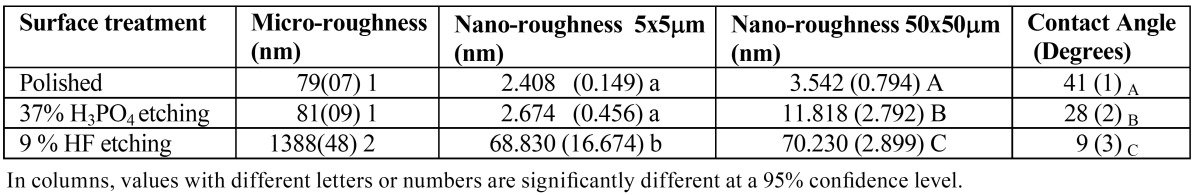
Mean and standard deviation (SD) of the IPS Empress surface micro- (Ra), nano-roughness (sRa) and contact angles (degrees) after different surface treatments studied.

Water contact angle results are summarized in ( Table 2). HF and phosphoric acid treatments improved significantly the ceramic surface wettability, decreasing the water contact angle. Differences existed between both treatments. HF produced the highest value.

Surface treatments affected significantly the SBS produced by all the adhesive systems used in this study (p<0.001). HF treated group showed the highest SBS to the resin composite (SBMP: 4.16±2.7 MPa; Clearfil: 5.18±3.3 MPa; ProBond: 4.05±3.1 MPa). More cohesive failures were recorded for HF groups (SBMP: 89%, Clearfil: 95% and Probond: 90%), usually associated with higher SBS values. Phosphoric acid groups exhibited the lowest SBS (SBMP: 11.04±3.3; Clearfil: 9.12±4.0; ProBond: 4.05±3.1) and highest percentage of adhesive failures (SBMP: 40%, Clearfil: 50% and Probond: 34%). No differences were produced by the different adhesive systems with any surface treatments evaluated.

**Figure 2:**
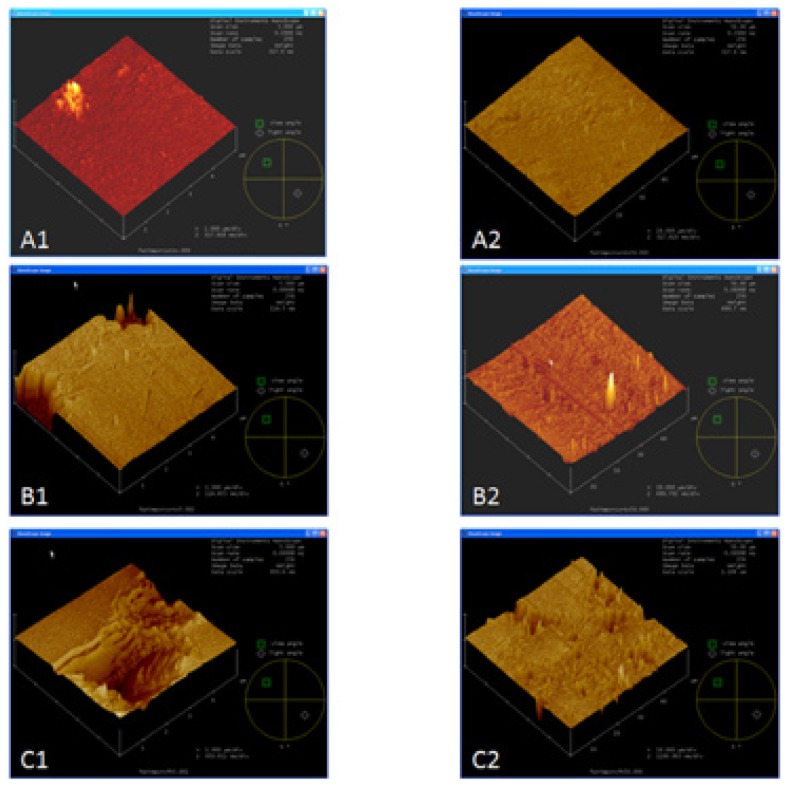
AFM images of ceramic surfaces conditioned with the different treatments: A) no treatment, B) 37% phosphoric acid for 15 s., C) 9% HF for 5 min. At different scan sizes: 1) 5x5 µm and 2) 50x50 µm.

Representative AFM images are reported in (Fig. 2) (5x5 µm and 50x50 µm). Phosphoric acid treatment (Fig. 2: B1) generated similar topography in the 5x5 µm to polished ceramic areas (Fig. 2: A1). Some micro-pores were rarely observed in phosphoric acid treated surface images (Fig. 2: B1). On the other hand, when 50x50 µm areas were examined, differences in topographies could be detected (Fig. 2: A2 and B2). After hydrofluoric acid treatment, ceramic surface exhibited the deepest irregularities (Fig. 2: C1 and C2).

**Figure 3:**
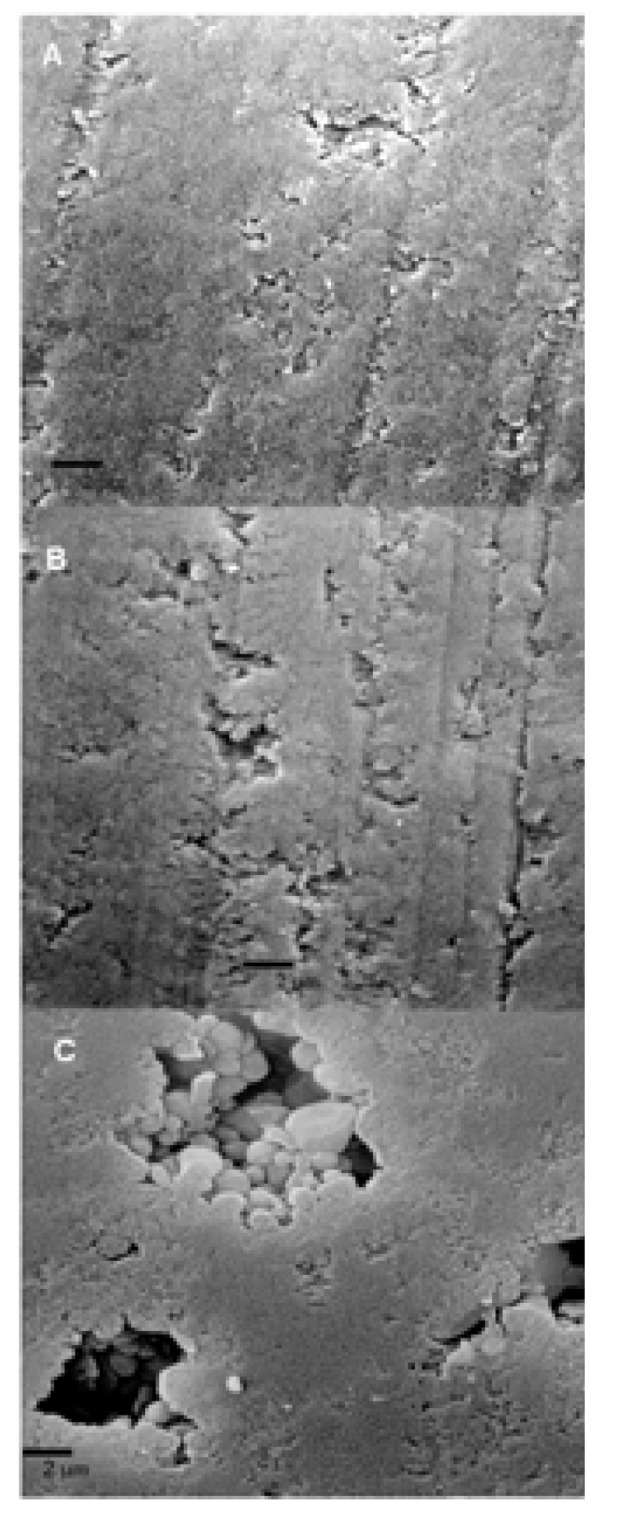
SEM images (5000x) of the ceramic surfaces conditioned with the different treatments: A) no treatment, B) 37% phosphoric acid for 15 s., C) 9% HF for 5 min.

SEM images are observed in (Fig. 3). The polished ceramic surface is showed with some scratches from the polish procedure (Fig. 3A). Phosphoric treated surface (Fig. 3B) exhibit some small grooves. Finally, HF treated surface (Fig. 3C) reveals a different irregular pattern with deep pores produced by the conditioner agent.

## Discussion

Ceramic restorations provide high biocompatibility and excellent aesthetic characteristics in dentistry (14). Glass ceramics are polycrystalline solids which are prepared by the controlled crystallization of glasses. These ceramic materials are performed by a leucite crystals precipitation in a glassy matrix. Leucite is the main crystal phase of the glass-ceramic (14). A strong resin bond relies on micro mechanical interlocking and chemical bonding to the ceramic surface, which requires roughening and cleaning for adequate surface activation. Common treatment options involve acid etching with solutions of hydrofluoric acid, phosphoric acid, nitric acid and others. Hydrofluoric acid has been found to be effective for improving bond strengths to ceramic (15).

It was discussed whether bond strength tests are, in general, able to predict the clinical behavior of adhesively bonded composite resin restorations since they do not represent the complex clinical failure mechanism (16). However, laboratory testing can make clear the specific factors that are most detrimental to long-term bonding effectiveness. Clinical trials are expensive and time consuming; therefore preclinical laboratory tests may be used for screening purposes.

In the present study, Watanabe jibs were used to evaluate the shear bond strength. The stresses at the restoration interface are complex, but they can be described as mainly a tensile or shear type of stress (17) [15] G. Oilo, Bond strength testing—what does it mean?, Int Dent J 43 (1993), pp. 492–498. View Record in Scopus Cited By in Scopus (74). Accordingly, methods for determining tensile bond strength and those for shear bond strength have been published. In tensile tests, e.g. the classical tensile bond strength test or the μ-tensile bond strength test (μTBS), the fracture starts at the weakest part of the bond. A disadvantage of these tests is the high technique sensitivity-micro fractures at the interface during specimen preparation may weaken the bond and reduce the actual bond strength (18). In a shear test the fracture does not start at the weakest part of the bond, but always at the insertion point of the load (19). Advantages of the shear bond test are the comparatively simple test procedure and the reproducible starting point of loading (19). Therefore, the shear bond test was included into the ISO/TS 11405:2003 (ISO/TS 11405:2003 (E). The ISO/TS 11405:2003 were followed in previous studies to test the bond efficacy (20,21).

The null hypothesis of this study was rejected. Significant differences among the groups were found in 3D nano-roughness which were not encountered in 2D micro-roughness. CA tested and SBS results were in accord with 3D nano-roughness measurements. HF treatment improved wettability on ceramic surfaces. Chemical etching selectively dissolves the glassy matrix and crystals in ceramics, generating an irregular topography with microretentive channels (22). In our study, HF treatment proved to be the most effective and reliable surface treatment associated with the increase of bond strength with a composite resin. Obtained bond strength values may have been attributed to the increased surface roughness and irregularities associated with hydrofluoric acid etching (Fig. 3C and Fig. 2: C1 and C2). HF conditioned surface exhibited the highest micro- and nano-roughness values ( Table 2), as resulted in previous studies. (22) Our data showed that phosphoric acid did not modify the roughness. Only in 50 x 50 μm AFM images, roughness values exhibited discrepancies between the phosphoric acid treated surfaces and the polished ones (Fig. 2: A2 and B2). The results showed that 2D micro-roughness did not present differences that 3D nanoroughness did. The average roughness (Ra) has been the only parameter used to assess the ceramic surface topography. Ra measures the average length between peaks and valleys and the deviation from the mean line on the entire surface within the studying length (23,24). Ra is a good parameter to describe the height variation, but is insensitive to wavelength and occasional high peaks and low valleys (23). In the present study, the Ra values assessed by profilometer among polished and phosphoric acid treated ceramic surface is simi-lar, although the morphology of these surfaces was considerably different as shown by SEM (Figs. 3A and 3B). The acid etching imposed an additional micro-roughness onto the previous roughness produced by the polish procedure (Fig. 3A and 3B). So, the current results confirmed that Ra may not be sufficient to describe surface morphology.

The 2D micro-roughness examination is deficient to explain correctly the surface topography of ceramic. It must be analyzed using the amplitude, the frequency and other parameters describing the organization topography (25). This poses a limitation of the profilometer (24). The important factor that profilometer do not assess is the skewness. This parameter is used to describe the shape of the topography height distribution. A surface with symmetrical shape for the surface height distribution, the skewness is zero. For an asymmetric distribution of surface heights, the skewness may be negative or positive (24).

On the other hand, the physics of the measuring instrument have an effect on the data. For contact measurement on ceramic surface, the probe may be too blunt to reach the bottom of deep valley and it may round the tips of sharp peaks. AFM uses a nanometric probe that facilitates the scan of nanometric details on the surface. The 3D parameter is sRa, which generalizes the calculus along two axes (26). An area rather than a line is scanned in AFM (3D). Although, AFM verified a smaller area, it was representative, because it scanned point-to-point and covered the whole. The roughness is scale-dependent and increases when larger area is studied (27). The possibility to find surfaces irregularities increases when the studied area growths (Figs. 1 and 2) (28). Roughness is a mixture of topographical features of several different lateral and vertical dimensions, and only when the scanning length is wide enough to include the largest ones, the roughness parameters begin to remain independent of the scanning length area (8). This could explain why these differences among polished and phosphoric acid treated ceramic surfaces were not found in 5 x 5 microns images (Fig. 2: A1 and B1).

In the present study, the phosphoric acid exhibited a lower effect on the ceramic surface than HF. This is in agreement with others authors (3). IPS Empress framework consists of a leucite reinforced glass ceramic from the K2O-Al2O3-SiO2 system. Chemical etching selectively dissolves the glassy matrix. This inter granular (inter-crystals) corrosion may be, partially, suppressed by the fast formation of an insoluble AlPO4 salt. This salt would protect the ceramic surface against further acid attack. Therefore, the penetration depths are small (29).These data were also supported by SEM and AFM evaluation (Fig. 2: B1 and B2, Fig. 3B), which portrayed that phosphoric acid treatment did not produce a retentive surface topography on the ceramic specimens, in comparison with the hydrofluoric acid treated group. El Zohairy et al. (2) found in SEM examinations that phosphoric acid is limited to clean the porcelain surface and it only exposed surface porosities and defects without any apparently etching pattern. This confirms the result of several previous studies (3).

Water contact angles were significantly different in all the studied groups ( Table 2). HF treated surfaces showed the lowest CA. Phosphoric acid group exhibited lower CA than the polished ones. This agreed with 3D nano-roughness results in 50x50 µm scan sizes. Oh and Shen (23) also obtained a reduction in CA after HF and phosphoric acid etching. Phoenix & Shen (6) showed the smallest contact angle on ceramic substrates after etching with HF, although with different data, probably due to the use of different methods (3). In the present study, the CA results are related to the 3D nano-roughness exhibited by each type of treated surfaces. Wetting of the ceramic with a liquid was significantly affected by the roughness of the ceramic surface. Water contact angle studies exhibited different wetting behaviors on the roughened ceramic surfaces (22). Roughness of substrate influenced the contact angle as a function of the Wenzel equation (30): r = cosθ1/ cosθ2

In which r gives the ratio of actual to apparent or projected area, and contact angles θ1 and θ2 refer to the roughened surface and true (smooth surface) contact angles, respectively.

SBS of HF-treated ceramic surfaces was higher than when phosphoric acid was employed. This highly micro mechanical retentive topography leads to an increase of the surface area, thus improving the possibility of micro-mechanical attachment with the resin. This complies with the biggest percentage of cohesive failure that the HF groups showed. In the present study, 3D nano-roughness results are confirmed.

No differences were shown in SBS values between the adhesive systems used in any of the surface treatments tested. The bond of ceramic to resin composite is usually created by two complementary mechanisms: micro-mechanical attachment (by mean of roughness) and chemical bonding (14). The establishment of a strong chemical bond between the dental ceramic and resin composite can be improved by treatment with a silane coupling agent.

No previous studies have been found concerning the common use of all these important variables to evaluate the dental ceramic adhesion: nano and micro-roughness, wettability and shear bond strength to composite resin. Within the limitation of this study, it is concluded that 3D nano-roughness evaluation was the most efficient tool to evaluate the topographical changes produced by the different acid treatments on ceramic surfaces.

## References

[1] Kumbuloglu O, Lassila LV, User A, Vallittu PK (2006). Bonding of resin composite luting cements to zirconium oxide by two air-particle abrasion methods. Oper Dent.

[2] El Zohairy AA, De Gee AJ, Hassan FM, Feilzer AJ (2004). The effect of adhesives with various degrees of hydrophilicity on resin ceramic bond durability. Dent Mater.

[3] Pisani-Proença J, Erhardt MC, Valandro LF, Gutierrez-Aceves G, Bolaños-Carmona V, Del Castillo-Salmeron R (2006). Influence of ceramic surface conditioning and resin cements on microtensile bond strength to a glass ceramic. J Prosthet Dent.

[4] Ayad MF, Fahmy NZ, Rosenstiel SF (2008 ). Effect of surface treatment on roughness and bond strength of a heat-pressed ceramic. J Prosthet Dent.

[5] Panah FG, Rezai SM, Ahmadian L (2008). The influence of ceramic surface treatments on the micro-shear bond strength of composite resin to IPS Empress 2. J Prosthodont.

[6] Phoenix RD, Shen C (1995). Characterization of treated porcelain surfaces via dynamic contact angle analysis. Int J Prosthodont.

[7] Erickson RL (1992). Surface interactions of dentin adhesive materials. Oper Dent.

[8] Marshall SJ, Bayne SC, Baier R, Tomsia AP, Marshall GW (2010). A review of adhesion science. Dent Mater.

[9] Méndez-Vilas A, Bruque JM, González-Martín ML (2007). Sensitivity of surface roughness parameters to changes in the density of scannig points in multi-scale AFM studies. Application to a biomaterial surface. Ultramicroscopy.

[10] Toledano M, Osorio E, Aguilera FS, Gomes G, Perdigão J, Osorio R (2010). Bond strength and nanoroughness assessment on human pretreated cementum surfaces. J Dent.

[11] Sano H, Shono T, Sonoda H, Takatsu T, Ciucchi B, Carvalho R (1994). Relationship between surface area for adhesion and tensile bond strength--evaluation of a micro-tensile bond test. Dent Mater.

[12] Scherrer SS, Cesar PF, Swain MV (2010). Direct comparison of the bond strength results of the different test methods: A critical literature review. Dent Mater.

[13] Braga RR, Meira JB, Boaro LC, Xavier TA (2010). Adhesion to tooth structure: A critical review of “macro” test methods. Dent Mater.

[14] Blatz MB, Sadan A, Kern M (2003). Resin-ceramic bonding: a review of the literature. J Prosthet Dent.

[15] Anusavice KJ (1992). Degradability of dental ceramics. Adv Dent Res.

[16] Van Meerbeek B, DeMunck J, Yoshida Y, Inoue S, Vargas M, Vijay P (2003). Buonocore memorial lecture. Adhesion to enamel and dentin: current status and future challenges, Oper Dent.

[17] Dietschi D, Herzfeld D (1998). In vitro evaluation of marginal and internal adaptation of class II resin composite restorations after thermal and occlusal stressing. Eur J Oral Sci.

[18] Oilo G (1993). Bond strength testing—what does it mean? Int Dent J.

[19] Goracci C, Sadek FT, Monticelli F, Cardoso PE, Ferrari M (2004). Influence of substrate, shape, and thickness on microtensile specimens' structural integrity and their measured bond strengths. Dent Mater.

[20] Watanabe I, Nakabayashi N (1994 ). Measurement methods for adhesion to dentine: the current status in Japan. J Dent.

[21] Osorio R, Ceballos L, Tay F, Cabrerizo-Vilchez MA, Toledano M (2002). Effect of sodium hypochlorite on dentin bonding with a polyalkenoic acid-containing adhesive system. J Biomed Mater Res.

[22] Toledano M, Perdigão J, Osorio E, Osorio R (2002). Influence of NaOCl deproteinization on shear bond strength in function of dentin depth. Am J Dent.

[23] Oh WS, Shen C (2003 ). Effect of surface topography on the bond strength of a composite to three different types of ceramic. J Prosthet Dent.

[24] Wennerberg A, Hallgren C, Johansson C, Danelli S (1998). A histomorphometric evaluation of screw-shaped implants each prepared with two surface roughnesses. Clin Oral Implants Res.

[25] Canabarro A, Figueiredo F, Paciornik S, De-Deus G (2009). Two- and three dimensional profilometer assessments to determine titanium roughness. Scanning.

[26] Bigerelle M, Anselme K, Noël B, Ruderman I, Hardouin P, Iost A (2002 ). Improvement in the morphology of Ti-based surfaces: a new process to increase in vitro human osteoblast response. Biomaterials.

[27] Méndez-Vilas A, Bruque JM, González-Martín ML (2007). Sensitivity of surface roughness parameters to changes in the density of scanning points in multi-scale AFM studies. Application to a biomaterial surface. Ultramicroscopy.

[28] Tholt de Vasconcellos B, Miranda-Júnior WG, Prioli R, Thompson J, Oda M (2006). Surface roughness in ceramics with different finishing techniques using atomic force microscope and profilometer. Oper Dent.

[29] Jardel V, Degrange M, Picard B, Derrien G (1999). Surface energy of etched ceramic. Int J Prosthodont.

[30] Marshall SJ, Bayne SC, Baier R, Tomsia AP, Marshall GW (2010). A review of adhesion science. Dent Mater.

